# Supplementary material for: Leveraging multinational enterprises to reduce the escalating regional carbon inequality in China
Source: Nat Commun. 2025 Jul 17;16:6603. doi: 10.1038/s41467-025-61968-8 (PMC12271316; doi:10.1038/s41467-025-61968-8)
Supplement: Supplementary file 1 — Supplementary information [file 41467_2025_61968_MOESM1_ESM.pdf]

Supplementary Information for

Leveraging multinational enterprises to reduce the escalating regional carbon inequality in China

Kailan Tian[1,2,#], Yu Zhang[3,#], Jing Meng[4,*], Zhuoying Zhang[5,*], Yuli Shan[6], Heran Zheng[4], Xiaowei Nie[5,7], Cuihong Yang[1,2,*]

1. State Key Laboratory of Mathematical Sciences, Academy of Mathematics and Systems Science, Chinese Academy of Sciences, Beijing 100190, China
2. School of Economics and Management, University of Chinese Academy of Sciences, Beijing 100190, China
3. Yangtze Industrial Economic Institute, Nanjing University, Nanjing 210093, China
4. The Bartlett School of Sustainable Construction, University College London, London, WC1E 6BT, UK
5. National Tibetan Plateau Data Center, State Key Laboratory of Tibetan Plateau Earth System, Environment and Resources (TPESER), Institute of Tibetan Plateau Research, Chinese Academy of Sciences, Beijing 100101, China
6. School of Geography, Earth and Environmental Sciences, University of Birmingham, Birmingham, B15 2TT, UK
7. School of Ecology and Environment, Tibet University, Lhasa 850000, China

# These authors contributed equally: Kailan Tian, Yu Zhang.

* Corresponding authors: Jing Meng (jing.j.meng@ucl.ac.uk), Zhuoying Zhang (zhangzy@itpcas.ac.cn), Cuihong Yang (chyang@iss.ac.cn)

Supplementary Note 1. Data sources and description

Interprovincial input-output tables that distinguish MNEs activities

This study uses interprovincial input-output (IPIO) tables from a newly published dataset[1]. This novel IPIO table distinguishes three types of enterprises by ownership in each province-industry pair, which are mainland China-, Hong Kong, Macao, Taiwan-, and foreign-owned enterprises, respectively (see Table S1 for the stylized table). We use the tables for the year 1997, 2002, 2007, 2012, and 2017. The table covers 31 provincial units of the mainland China (Table S2) and 42 sectors in each province. We also aggregate the 31 provincial units into 4 zones for the purpose of illustration. The sector classifications are not consistent in the tables for different years. We thus aggregate some sectors and derive 30 sectors and we identify 6 carbon-intensive industries (Table S3) according to their high carbon emission coefficients.

Carbon emissions data

The sectoral CO_2 emission inventories are obtained from the CEADs database[2,3]. Emissions are calculated based on IPCC (Intergovernmental Panel on Climate Change) administrative territorial scope that covers all anthropogenic emissions generated within an administrative boundary. It is worth noting that there could be uncertainties in the CO_2 emission inventory. According to the database developers, factors such as the activity data, emission factors, lack of completeness, and measurement errors can introduce different levels of uncertainty. They use Monte Carlo simulation to assess the uncertainty in the emission data and update the emission data when new information becomes available. Despite these uncertainties, their estimates are considered reliable and are widely used by researchers.

The CEADs does not distinguish between sectoral carbon emissions from domestic-owned, HMT-owned and foreign-owned enterprises. Therefore, we need to disaggregate the sectoral carbon emissions. Previous studies provide three different approaches. The first approach estimates the carbon emissions of sub-sectors according to the intermediate input structure[4]. The second approach estimates them by

assuming that sub-sectors have the same carbon intensity[5] (carbon emissions per unit of value-added). The third approach assumes that sub-sectors have the same emission intensities as the representative regions[6]. In this study, we take the first approach and use the intermediate use of fuel resources to split sectoral emissions into three parts which are the emissions of domestic-owned, HMT-owned and foreign-owned enterprises, respectively. The assumption is that enterprises with greater fuel consumption emit higher amount of carbon.

Supplementary Note 2. Examining the effects of MNEs on emission efficiency

To examine the effects of MNEs on emission efficiency, we estimate a panel fixed effects model for industry i of province r at time t:

$$emf_{i,r,t} = \alpha MNE_{i,r,t} + \beta X + \mu_i + \mu_r + \mu_t + \varepsilon_{i,r,t} \ . \tag{1}$$

The emission efficiency ($emf_{i,r,t}$) is the dependent variable. We consider two distinct dependent variables: (i) a province-industry's emission coefficient (ec, emission per unit of economic output) and (ii) the ratio of the emission coefficient of domestic-owned enterprises to that of MNEs in the same industry (gap). The second indicator measures the emission efficiency gap between domestic-owned enterprises and MNEs. We take the explanatory variable ($MNE_{i,r,t}$) as the extent of MNEs' presence in an industry, which is measured by the ratio of the industry's intermediate consumption from MNEs to its overall intermediate consumption.

We use instrumental variables to address the possibility that endogeneity and reverse causality may lead to a biased result. For instance, industries with inherently lower emission intensities might naturally attract more MNEs due to factors such as alignment with global environmental standards or the industry's higher technology and service-oriented focus. We consider two instrumental variables. The first is the Foreign Market Access (FMA). A province's FMA is measured by the distance from its capital city to the nearest port. Provinces closer to a port are more likely to have better access to foreign markets, thereby making them more appealing to MNEs. In

other words, there is a correlation between FMA and the presence of MNEs (the explanatory variable). Furthermore, a region's emission efficiency (the dependent variable) is not directly correlated with its geographical characteristics. Therefore, this variable is well-suited for the IV role because geographic characteristics are unlikely to be influenced by other economic or social factors. The second is the commonly used one-period (5 years in our case) lagged variable ($MNE_{i,r,t-5}$). Lagged variables are frequently employed as IVs because they are often correlated with the current values of the explanatory variable while being less likely to be influenced by contemporaneous shocks or reverse causality. We also check the robustness of the results by employing an alternative estimator-GMM to partially solve the endogeneity problem.

The main channels through which a region's emission performance is determined are the scale effects, the sectoral composition effects, and the technique effects[7]. We thus include control variables to capture such effects. Our control variables include the economic scale ($sca_{i,r,t}$), which is the value-added of industry i in province r at time t; the industrial structure ($str_{r,t}$), measured by the value-added share of province r's secondary industry in the province's total value-added; technology innovation level ($tec_{r,t}$), proxied by the ratio of province r's research and development (R&D) investment to the province's GDP; urbanization level ($urb_{r,t}$); environmental regulation level ($reg_{r,t}$). The Chinese government set energy intensity reduction targets for every province in the country's tenth (2001-2005), eleventh (2006-2010), twelfth (2011-2015), thirteenth (2016-2020) Five-Year Plans. We use these reduction targets as a proxy for the environmental regulation level. We also use the province-level environmental regulation index from Lin et al.[8] as an alternative proxy and derive robust results. Their index is calculated using the weighted value of five indicators: the proportion of sulfur dioxide removed, the proportion of smoke (dust) removed, the proportion of wastewater that meets discharge standards, the ratio of solid wastes utilized, and the ratio of household wastes harmlessly treated. The weight of each indicator is determined by information entropy.

We include a set of industry fixed effects (μ_i), province fixed effects (μ_r) and time fixed effects (μ_t) to account for possible country, industry and time unobserved heterogeneity, and thus, we argue that omitted variables bias is not a (big) concern. We would further like to stress that our main goal is to examine the association between the presence of MNEs and emission performance, rather than causal effects.

Supplementary Note 3.

Method robustness check: the hypothetical extraction method and the decomposition method

The traditional IO Leontief model enables us to trace the value added and CO_2 emissions generated by MNEs' outputs that are used to satisfy final demand. However, it does not allow us to trace the value added or CO_2 emissions embodied in MNEs' outputs used as intermediate inputs simply by multiplying the Leontief inverse and the intermediate inputs. Therefore, previous studies on global value chain (GVC) accounting have proposed the IO-based decomposition method[9] and the hypothetical extraction method[10] (HEM) to address this issue. Although the two methods yield the same results, Los et al.[10] note that the decomposition method is overly complex in terms of both mathematical derivation and interpretation. They thus propose the more intuitive HEM, which can be more easily applied to the data. Next, we mathematically prove that the HEM and the decomposition method yield the same results. We derive the results for CO_2 emissions, and the results for value added can be obtained in the same way.

Let $\mathbf{e}$ be the direct carbon emission coefficient vector, and $\mathbf{Y}$ be final demand. The CO_2 emissions generated by final demand can be written as

$$\mathrm{c} = \mathbf{e}(\mathbf{I} - \mathbf{A})^{-1}\mathbf{Y}. \qquad (2)$$

Taking region r as an example, we define $\mathbf{Y}_r^F$ as the supply of its MNEs' outputs for satisfying final demand and $\mathbf{Z}_r^F = \mathbf{A}_r^F\mathbf{x}$ as the activities related to its MNEs' intermediate production, respectively, with

$$\mathbf{Y}_r^F=\begin{bmatrix} \mathbf{0} & \mathbf{0} & \cdots & \mathbf{0} \\ \mathbf{0} & \mathbf{0} & \cdots & \mathbf{0} \\ \mathbf{0} & \mathbf{0} & \cdots & \mathbf{0} \\ \mathbf{Y}_{r1}^F & \mathbf{Y}_{r2}^F & \cdots & \mathbf{Y}_{rm}^F \\ \vdots & \vdots & \vdots & \vdots \\ \mathbf{0} & \mathbf{0} & \cdots & \mathbf{0} \end{bmatrix}, \text{ and } \mathbf{A}_r^F=\begin{bmatrix} \mathbf{0} & \mathbf{0} & \cdots & \mathbf{A}_{1r}^{DF} & \mathbf{0} & \mathbf{0} \\ \mathbf{0} & \mathbf{0} & \cdots & \mathbf{A}_{1r}^{FF} & \mathbf{0} & \mathbf{0} \\ \mathbf{0} & \mathbf{0} & \cdots & \vdots & \mathbf{0} & \mathbf{0} \\ \mathbf{A}_{r1}^{FD} & \mathbf{A}_{r1}^{FF} & \cdots & \mathbf{A}_{rr}^{FF} & \mathbf{A}_{rm}^{FD} & \mathbf{A}_{rm}^{FF} \\ \vdots & \vdots & \vdots & \vdots & \vdots & \vdots \\ \mathbf{0} & \mathbf{0} & \cdots & \mathbf{A}_{mr}^{FF} & \mathbf{0} & \mathbf{0} \end{bmatrix}.$$

We further have $\mathbf{Y}_r^{F*} = \mathbf{Y} - \mathbf{Y}_r^F$, and $\mathbf{A}_r^{F*} = \mathbf{A} - \mathbf{A}_r^F$.

Hypothetical extraction method

The CO_2 emissions generated by MNEs in region r can be measured by the difference between the original value and the hypothetical value, which is calculated by extracting the production activities of MNEs in region r. That is

$$\begin{aligned}
c_r^F &= \mathbf{e}_r(\mathbf{I}-\mathbf{A})^{-1}\mathbf{Y} - \mathbf{e}_r(\mathbf{I}-\mathbf{A}_r^{F*})^{-1}\mathbf{Y}_r^{F*} \\
&= \mathbf{e}_r(\mathbf{I}-\mathbf{A})^{-1}\mathbf{Y} - \mathbf{e}_r\left(\mathbf{I}-\mathbf{A}+\mathbf{A}_r^F\right)^{-1}(\mathbf{Y}-\mathbf{Y}_r^F) \\
&= \mathbf{e}_r[\left(\mathbf{I}-\mathbf{A}+\mathbf{A}_r^F\right)^{-1}\left(\mathbf{I}-\mathbf{A}+\mathbf{A}_r^F\right)(\mathbf{I}-\mathbf{A})^{-1}\mathbf{Y} - \left(\mathbf{I}-\mathbf{A}+\mathbf{A}_r^F\right)^{-1}\left(\mathbf{Y}-\mathbf{Y}_r^F\right)] \\
&= \mathbf{e}_r[\left(\mathbf{I}-\mathbf{A}+\mathbf{A}_r^F\right)^{-1}\mathbf{Y} + \left(\mathbf{I}-\mathbf{A}+\mathbf{A}_r^F\right)^{-1}\mathbf{A}_r^F(\mathbf{I}-\mathbf{A})^{-1}\mathbf{Y} - \left(\mathbf{I}-\mathbf{A}+\mathbf{A}_r^F\right)^{-1}\mathbf{Y} \\
&\qquad + \left(\mathbf{I}-\mathbf{A}+\mathbf{A}_r^F\right)^{-1}\mathbf{Y}_r^F] \\
&= \mathbf{e}_r[(\mathbf{I}-\mathbf{A}+\mathbf{A}_r^F)^{-1}\mathbf{A}_r^F(\mathbf{I}-\mathbf{A})^{-1}\mathbf{Y} + (\mathbf{I}-\mathbf{A}+\mathbf{A}_r^F)^{-1}\mathbf{Y}_r^F] \\
&= \mathbf{e}_r(\mathbf{I}-\mathbf{A}+\mathbf{A}_r^F)^{-1}[\mathbf{A}_r^F(\mathbf{I}-\mathbf{A})^{-1}\mathbf{Y} + \mathbf{Y}_r^F] \\
&= \mathbf{e}_r(\mathbf{I}-\mathbf{A}+\mathbf{A}_r^F)^{-1}[\mathbf{A}_r^F\mathbf{x} + \mathbf{Y}_r^F] \qquad (3)
\end{aligned}$$

The decomposition method

Using the decomposition framework, we first need to decompose the CO_2 emissions in region r into three terms.

$$\begin{aligned}
c_r &= \mathbf{e}_r(\mathbf{I}-\mathbf{A})^{-1}\mathbf{Y} \\
&= \mathbf{e}_r\left(\mathbf{I}-\mathbf{A}_r^{F*}-\mathbf{A}_r^F\right)^{-1}(\mathbf{Y}_r^{F*}+\mathbf{Y}_r^F) \\
&= \mathbf{e}_r(\mathbf{I}-\mathbf{A}_r^{F*}-\mathbf{A}_r^F)^{-1}\mathbf{Y}_r^{F*} + \mathbf{e}_r(\mathbf{I}-\mathbf{A})^{-1}\mathbf{Y}_r^F \qquad (4)
\end{aligned}$$

Note that $\mathbf{I} = (\mathbf{I}-\mathbf{A}_r^{F*})^{-1}(\mathbf{I}-\mathbf{A}_r^{F*})$ and $\mathbf{I} = (\mathbf{I}-\mathbf{A}_r^{F*}-\mathbf{A}_r^F)^{-1}(\mathbf{I}-\mathbf{A}_r^{F*}-\mathbf{A}_r^F)$, we have

$$\begin{aligned}
(\mathbf{I}-\mathbf{A}_r^{F*}-\mathbf{A}_r^F)^{-1} &= (\mathbf{I}-\mathbf{A}_r^{F*})^{-1}(\mathbf{I}-\mathbf{A}_r^{F*}-\mathbf{A}_r^F+\mathbf{A}_r^F)(\mathbf{I}-\mathbf{A}_r^{F*}-\mathbf{A}_r^F)^{-1} \\
&= (\mathbf{I}-\mathbf{A}_r^{F*})^{-1}[\mathbf{I}+\mathbf{A}_r^F(\mathbf{I}-\mathbf{A})^{-1}].
\end{aligned}$$

Then, equation (4) can be rewritten as

$$\mathrm{c}_r = \mathbf{e}_r\left(\mathbf{I}-\mathbf{A}_r^{F*}\right)^{-1}\left[\mathbf{I}+\mathbf{A}_r^{F}(\mathbf{I}-\mathbf{A})^{-1}\right]\mathbf{Y}_r^{F*} + \mathbf{e}_r(\mathbf{I}-\mathbf{A})^{-1}\mathbf{Y}_r^{F}$$

$$= \mathbf{e}_r(\mathbf{I}-\mathbf{A}_r^{F*})^{-1}\mathbf{Y}_r^{F*} + \mathbf{e}_r(\mathbf{I}-\mathbf{A}_r^{F*})^{-1}\mathbf{A}_r^{F}(\mathbf{I}-\mathbf{A})^{-1}\mathbf{Y}_r^{F*} + \mathbf{e}_r(\mathbf{I}-\mathbf{A})^{-1}\mathbf{Y}_r^{F} \qquad (5)$$

In equation (5), we obtain three terms. The first term represents the CO_2 emissions in region r that are not related to MNEs' production activities; the second term gives the emissions generated by the output of MNEs that are used for intermediate production; the third term gives the emissions generated by the output of MNEs that are used for final demand. Therefore, the CO_2 emissions generated by MNEs in region r are the summation of the second and the third term.

$$\mathrm{c}_r^F = \mathbf{e}_r(\mathbf{I}-\mathbf{A}_r^{F*})^{-1}\mathbf{A}_r^{F}(\mathbf{I}-\mathbf{A})^{-1}\mathbf{Y}_r^{F*} + \mathbf{e}_r(\mathbf{I}-\mathbf{A})^{-1}\mathbf{Y}_r^{F}$$

$$= \mathbf{e}_r(\mathbf{I}-\mathbf{A}_r^{F*})^{-1}\mathbf{A}_r^{F}(\mathbf{I}-\mathbf{A})^{-1}\mathbf{Y}_r^{F*} + \mathbf{e}_r(\mathbf{I}-\mathbf{A}_r^{F*})^{-1}(\mathbf{I}-\mathbf{A}_r^{F*}-\mathbf{A}_r^{F}+\mathbf{A}_r^{F})(\mathbf{I}-\mathbf{A})^{-1}\mathbf{Y}_r^{F}$$

$$= \mathbf{e}_r(\mathbf{I}-\mathbf{A}_r^{F*})^{-1}\mathbf{A}_r^{F}(\mathbf{I}-\mathbf{A})^{-1}\mathbf{Y}_r^{F*} + \mathbf{e}_r(\mathbf{I}-\mathbf{A}_r^{F*})^{-1}(\mathbf{I}-\mathbf{A}_r^{F*}-\mathbf{A}_r^{F})(\mathbf{I}-\mathbf{A})^{-1}\mathbf{Y}_r^{F}$$
$$+ \mathbf{e}_r(\mathbf{I}-\mathbf{A}_r^{F*})^{-1}\mathbf{A}_r^{F}(\mathbf{I}-\mathbf{A})^{-1}\mathbf{Y}_r^{F}$$

$$= \mathbf{e}_r(\mathbf{I}-\mathbf{A}_r^{F*})^{-1}\mathbf{A}_r^{F}(\mathbf{I}-\mathbf{A})^{-1}\mathbf{Y}_r^{F*} + \mathbf{e}_r(\mathbf{I}-\mathbf{A}_r^{F*})^{-1}\mathbf{Y}_r^{F}$$
$$+ \mathbf{e}_r(\mathbf{I}-\mathbf{A}_r^{F*})^{-1}\mathbf{A}_r^{F}(\mathbf{I}-\mathbf{A})^{-1}\mathbf{Y}_r^{F}$$

$$= \mathbf{e}_r(\mathbf{I}-\mathbf{A}_r^{F*})^{-1}\mathbf{A}_r^{F}(\mathbf{I}-\mathbf{A})^{-1}\mathbf{Y} + \mathbf{e}_r(\mathbf{I}-\mathbf{A}_r^{F*})^{-1}\mathbf{Y}_r^{F}$$

$$= \mathbf{e}_r(\mathbf{I}-\mathbf{A}_r^{F*})^{-1}\mathbf{A}_r^{F}\mathbf{x} + \mathbf{e}_r(\mathbf{I}-\mathbf{A}_r^{F*})^{-1}\mathbf{Y}_r^{F}$$

$$= \mathbf{e}_r(\mathbf{I}-\mathbf{A}_r^{F*})^{-1}(\mathbf{A}_r^{F}\mathbf{x} + \mathbf{Y}_r^{F})$$

$$= \mathbf{e}_r(\mathbf{I}-\mathbf{A}+\mathbf{A}_r^{F})^{-1}(\mathbf{A}_r^{F}\mathbf{x} + \mathbf{Y}_r^{F}) \qquad (6)$$

We can see that equation (6) obtained by the decomposition method is the same as equation (3) derived from the HEM.

Supplementary Table 1. The environmentally extended interprovincial input-output model distinguishing three types of enterprises by ownership

		Intermediate use							Final demand			export	Gross output
		Region s			⋯	Region r			s	⋯	r		
		D	H	F	⋯	D	H	F					
Region s	D	$\mathbf{Z}_{ss}^{DD}$	$\mathbf{Z}_{ss}^{DH}$	$\mathbf{Z}_{ss}^{DF}$	⋯	$\mathbf{Z}_{sr}^{DD}$	$\mathbf{Z}_{sr}^{DH}$	$\mathbf{Z}_{sr}^{DF}$	$\mathbf{F}_{ss}^{D}$	⋯	$\mathbf{F}_{sr}^{D}$	$\mathbf{e}_{s}^{D}$	$\mathbf{y}_{s}^{D}$
	H	$\mathbf{Z}_{ss}^{HD}$	$\mathbf{Z}_{ss}^{HH}$	$\mathbf{Z}_{ss}^{HF}$	⋯	$\mathbf{Z}_{sr}^{HD}$	$\mathbf{Z}_{sr}^{HH}$	$\mathbf{Z}_{sr}^{HF}$	$\mathbf{F}_{ss}^{H}$	⋯	$\mathbf{F}_{sr}^{H}$	$\mathbf{e}_{s}^{H}$	$\mathbf{y}_{s}^{H}$
	F	$\mathbf{Z}_{ss}^{FD}$	$\mathbf{Z}_{ss}^{FH}$	$\mathbf{Z}_{ss}^{FF}$	⋯	$\mathbf{Z}_{sr}^{FD}$	$\mathbf{Z}_{sr}^{FH}$	$\mathbf{Z}_{sr}^{FF}$	$\mathbf{F}_{ss}^{F}$	⋯	$\mathbf{F}_{sr}^{F}$	$\mathbf{e}_{s}^{F}$	$\mathbf{y}_{s}^{F}$
⋮	⋮	⋮	⋮	⋮	⋮	⋮	⋮	⋮	⋮	⋮	⋮	⋮	⋮
Region r	D	$\mathbf{Z}_{rs}^{DD}$	$\mathbf{Z}_{rs}^{DH}$	$\mathbf{Z}_{rs}^{DF}$	⋯	$\mathbf{Z}_{rr}^{DD}$	$\mathbf{Z}_{rr}^{DH}$	$\mathbf{Z}_{rr}^{DF}$	$\mathbf{F}_{rs}^{D}$	⋯	$\mathbf{F}_{rr}^{D}$	$\mathbf{e}_{r}^{D}$	$\mathbf{y}_{r}^{D}$
	H	$\mathbf{Z}_{rs}^{HD}$	$\mathbf{Z}_{rs}^{HH}$	$\mathbf{Z}_{rs}^{HF}$	⋯	$\mathbf{Z}_{rr}^{HD}$	$\mathbf{Z}_{rr}^{HH}$	$\mathbf{Z}_{rr}^{HF}$	$\mathbf{F}_{rs}^{H}$	⋯	$\mathbf{F}_{rr}^{H}$	$\mathbf{e}_{r}^{H}$	$\mathbf{y}_{r}^{H}$
	F	$\mathbf{Z}_{rs}^{FD}$	$\mathbf{Z}_{rs}^{FH}$	$\mathbf{Z}_{rs}^{FF}$	⋯	$\mathbf{Z}_{rr}^{FD}$	$\mathbf{Z}_{rr}^{FH}$	$\mathbf{Z}_{rr}^{FF}$	$\mathbf{F}_{rs}^{F}$	⋯	$\mathbf{F}_{rr}^{F}$	$\mathbf{e}_{r}^{F}$	$\mathbf{y}_{r}^{F}$
Import		$\mathbf{M}_{s}^{D}$	$\mathbf{M}_{s}^{H}$	$\mathbf{M}_{s}^{F}$		$\mathbf{M}_{r}^{D}$	$\mathbf{M}_{r}^{H}$	$\mathbf{M}_{r}^{F}$	$\mathbf{M}_{s}$	⋯	$\mathbf{M}_{r}$		
Value added		$(\mathbf{w}_{s}^{D})'$	$(\mathbf{w}_{s}^{H})'$	$(\mathbf{w}_{s}^{F})'$	⋯	$(\mathbf{w}_{s}^{D})'$	$(\mathbf{w}_{s}^{H})'$	$(\mathbf{w}_{s}^{F})'$					
Gross input		$(\mathbf{y}_{s}^{D})'$	$(\mathbf{y}_{s}^{H})'$	$(\mathbf{y}_{s}^{F})'$	⋯	$(\mathbf{y}_{r}^{D})'$	$(\mathbf{y}_{r}^{H})'$	$(\mathbf{y}_{r}^{F})'$					
Carbon emission		$(\mathbf{c}_{s}^{D})'$	$(\mathbf{c}_{s}^{H})'$	$(\mathbf{c}_{s}^{F})'$	⋯	$(\mathbf{c}_{r}^{D})'$	$(\mathbf{c}_{r}^{H})'$	$(\mathbf{c}_{r}^{F})'$					

Supplementary Table 2 List of sectors

No.	Carbon intensity	Description
1		Farming, Forestry, Animal Production and Fishery
2	carbon-intensive	Mining, Extraction of Crude Petroleum and Natural Gas
3		Manufacture of Food and Tobacco
4		Manufacture of Textiles, Wearing, Apparel, Footwear, Leather, Fur, Feather and Its Products
5		Processing of Timbers and Manufacture of Furniture, Papermaking, Printing and Manufacture of Articles for Culture, Education and Sports Activities
6	carbon-intensive	Manufacture of Refined Petroleum, Coke Products, Processing of Nuclear Fuel
7	carbon-intensive	Manufacture of Chemicals and Chemical Products
8	carbon-intensive	Manufacture of Nonmetallic Mineral Products
9	carbon-intensive	Manufacture and Processing of Metals, Fabricated Metal Products,
10		Manufacture of General-Purpose Machinery and Special-Purpose Machinery
11		Manufacture of Transport Equipment
12		Manufacture of Electrical Machinery and Apparatus
13		Manufacture of Communication Equipment, Computer and Other Electronic Equipment
14		Manufacture of Measuring Instruments
15		Other Manufacture, Scrap and Waste
16	carbon-intensive	Production and Supply of Electricity,Gas, and Water
17		Construction
18		Wholesale and Retail Trade
19	carbon-intensive	Transport, Storage and Post
20		Accommodation, Food and Beverage Services
21		Information Transmission, Software and Information Technology Services
22		Finance
23		Real Estate
24		Renting and Leasing, Business Services
25		Research and Comprehensive Technical Services
26		Other Services
27		Education
28		Health Care and Social Work Activities
29		Culture, Sports and Entertainment
30		Public Management, Social Security and Social Organization

Note: The seven carbon-intensive sectors are identified according to their high carbon emission coefficient (carbon emission per unit of economic output).

Supplementary Table 3 Contribution of MNEs to provincial value-added (1997 & 2002 & 2007 &2012 &2017) (Unit: billion Yuan)

Regions	Provincial-level administrative entities	1997					
		Domestically-owned	HMT-owned	Foreign-owned	MNEs	Share of direct value-added of MNEs in local value-added	Contribution of MNEs to local value-added
Eastern China	Beijing	158	26	37	63	12.2%	28.4%
	Tianjin	83	15	34	49	19.6%	37.0%
	Hebei	318	30	34	63	3.2%	16.6%
	Shanghai	218	50	93	143	20.4%	39.6%
	Jiangsu	557	74	67	140	2.8%	20.1%
	Zhejiang	382	56	52	108	7.4%	22.0%
	Fujian	170	88	41	130	22.3%	43.3%
	Shandong	574	35	72	108	4.2%	15.8%
	Guangdong	543	183	87	270	22.2%	33.2%
	Hainan	38	3	3	5	3.3%	12.0%
North-eastern China	Liaoning	292	29	54	82	5.7%	22.0%
	Jilin	127	7	19	26	7.8%	17.2%
	Heilongjiang	215	15	20	35	2.4%	14.1%
Central China	Shanxi	128	11	15	26	2.2%	16.9%
	Anhui	227	12	20	33	2.3%	12.6%
	Jiangxi	153	9	6	15	3.9%	8.8%
	Henan	377	22	23	44	2.4%	10.5%
	Hunan	262	15	21	36	2.9%	12.2%
	Hubei	260	19	19	38	2.3%	12.7%
Western China	Inner Mongolia	108	7	6	13	1.8%	10.5%
	Guangxi	168	9	13	22	2.8%	11.4%
	Chongqing	134	10	16	25	4.5%	16.0%
	Sichuan	306	12	20	32	3.4%	9.4%
	Guizhou	77	3	4	7	0.5%	8.3%
	Yunnan	160	6	9	15	1.8%	8.6%
	Shaanxi	125	7	10	17	2.5%	11.9%
	Gansu	74	4	4	8	1.2%	10.2%
	Qinghai	19	1	1	2	0.8%	11.0%
	Ningxia	21	1	1	2	4.1%	9.6%
	Xinjiang	98	5	6	10	1.2%	9.6%
Regions	Provincial-level administrative entities	2002					
		Domestically-owned	HMT-owned	Foreign-owned	MNEs	Share of direct value-added of MNEs in local	Contribution of MNEs to local value-added
Eastern China	Beijing	285	58	117	174	16.0%	38.0%
	Tianjin	114	20	61	81	20.2%	41.5%
	Hebei	446	46	68	114	5.3%	20.4%
	Shanghai	281	93	214	307	25.0%	52.2%

	Jiangsu	661	156	258	415	15.9%	38.6%
	Zhejiang	570	113	132	245	10.4%	30.1%
	Fujian	243	109	101	210	19.9%	46.4%
	Shandong	803	62	158	219	7.9%	21.5%
	Guangdong	729	364	287	651	28.9%	47.2%
	Hainan	48	5	12	17	10.9%	25.8%
North-eastern China	Liaoning	356	64	134	198	12.7%	35.8%
	Jilin	162	14	31	45	5.9%	21.7%
	Heilongjian	256	22	51	73	2.9%	22.2%
Central China	Shanxi	206	10	20	30	2.7%	12.7%
	Anhui	315	28	46	73	4.3%	18.9%
	Jiangxi	216	16	17	33	3.8%	13.2%
	Henan	536	29	47	76	3.4%	12.4%
	Hunan	350	25	46	71	6.3%	17.0%
	Hubei	350	28	50	77	6.6%	18.1%
Western China	Inner Mongolia	171	8	18	26	3.0%	13.3%
	Guangxi	208	17	30	48	6.2%	18.6%
	Chongqing	179	19	33	53	7.1%	22.7%
	Sichuan	426	18	35	53	3.6%	11.0%
	Guizhou	113	5	9	14	1.9%	10.7%
	Yunnan	210	9	20	29	4.4%	12.1%
	Shaanxi	191	13	24	37	4.6%	16.3%
	Gansu	110	6	10	15	3.7%	12.2%
	Qinghai	32	1	2	3	3.4%	7.7%
	Ningxia	33	1	3	5	3.2%	12.4%
	Xinjiang	138	10	15	25	2.8%	15.3%

**Regions**	**Provincial-level administrative entities**	**2007**					
		**Domestically-owned**	**HMT-owned**	**Foreign-owned**	**MNEs**	**Share of direct value-added of MNEs in local**	**Contribution of MNEs to local value-added**
Eastern China	Beijing	652	136	252	388	18.7%	37.3%
	Tianjin	248	47	120	167	21.5%	40.3%
	Hebei	891	121	201	322	6.4%	26.6%
	Shanghai	532	216	538	753	32.4%	58.6%
	Jiangsu	1486	358	749	1107	21.1%	42.7%
	Zhejiang	1271	256	334	589	14.3%	31.7%
	Fujian	505	211	215	426	22.9%	45.7%
	Shandong	1779	113	375	488	7.2%	21.5%
	Guangdong	1628	807	732	1539	28.2%	48.6%
	Hainan	100	8	15	23	4.6%	18.5%
North-eastern China	Liaoning	780	67	181	247	10.7%	24.1%
	Jilin	301	27	79	106	8.6%	26.0%
	Heilongjian	472	42	98	139	4.2%	22.8%
Central China	Shanxi	508	27	58	84	2.7%	14.3%
	Anhui	611	64	117	181	4.4%	22.9%
	Jiangxi	429	72	75	147	8.8%	25.5%
	Henan	1227	110	142	252	4.0%	17.0%

	Hunan	799	54	73	127	2.9%	13.8%
	Hubei	767	60	116	176	6.3%	18.7%
Western China	Inner Mongolia	424	32	60	92	3.2%	17.8%
	Guangxi	421	47	78	125	6.7%	22.9%
	Chongqing	377	32	67	99	8.0%	20.7%
	Sichuan	920	42	91	134	4.4%	12.7%
	Guizhou	244	15	25	40	2.3%	14.2%
	Yunnan	432	29	46	75	2.7%	14.7%
	Shaanxi	446	40	81	121	3.9%	21.3%
	Gansu	239	9	19	28	2.1%	10.6%
	Qinghai	66	3	3	6	2.9%	7.9%
	Ningxia	73	4	11	15	3.2%	16.6%
	Xinjiang	292	25	33	58	1.2%	16.5%
**Regions**	**Provincial-level administrative entities**	**2012**					
		**Domestically-owned**	**HMT-owned**	**Foreign-owned**	**MNEs**	**Share of direct value-added of MNEs in local**	**Contribution of MNEs to local value-added**
Eastern China	Beijing	1302	204	380	584	18.4%	31.0%
	Tianjin	582	102	212	315	17.2%	35.1%
	Hebei	1929	139	220	359	3.8%	15.7%
	Shanghai	931	365	817	1182	34.0%	55.9%
	Jiangsu	3369	661	1296	1957	17.0%	36.7%
	Zhejiang	2408	469	533	1001	12.0%	29.4%
	Fujian	1182	450	370	820	18.3%	40.9%
	Shandong	3466	222	572	794	5.5%	18.6%
	Guangdong	3216	1226	1212	2438	24.2%	43.1%
	Hainan	223	24	30	54	5.8%	19.4%
North-eastern China	Liaoning	1357	132	281	413	9.9%	23.3%
	Jilin	716	54	90	144	5.7%	16.8%
	Heilongjiang	889	68	136	204	3.2%	18.6%
Central China	Shanxi	946	78	135	213	3.9%	18.4%
	Anhui	1482	126	211	337	3.7%	18.5%
	Jiangxi	1016	134	120	254	5.8%	20.0%
	Henan	2463	183	226	409	2.7%	14.3%
	Hunan	1867	95	142	236	2.8%	11.2%
	Hubei	1892	111	238	348	7.4%	15.6%
Western China	Inner Mongolia	881	55	103	158	1.6%	15.2%
	Guangxi	931	80	110	190	6.3%	16.9%
	Chongqing	895	91	163	254	7.6%	22.1%
	Sichuan	2066	121	186	306	5.1%	12.9%
	Guizhou	588	31	50	81	1.8%	12.1%
	Yunnan	962	53	85	138	2.8%	12.6%
	Shaanxi	1168	83	152	235	2.9%	16.7%
	Gansu	465	23	47	70	2.5%	13.1%
	Qinghai	143	4	5	8	0.8%	5.5%
	Ningxia	184	11	17	28	1.8%	13.2%
	Xinjiang	599	66	70	136	1.3%	18.5%

Regions	Provincial-level administrative entities	2017					
		**Domestically-owned**	**HMT-owned**	**Foreign-owned**	**MNEs**	**Share of direct value-added of MNEs in local**	**Contribution of MNEs to local value-added**
Eastern China	Beijing	2022	393	573	966	16.6%	32.3%
	Tianjin	771	186	288	474	19.0%	38.1%
	Hebei	2508	214	342	556	4.1%	18.2%
	Shanghai	1784	543	965	1508	26.2%	45.8%
	Jiangsu	5312	1196	2079	3275	14.7%	38.1%
	Zhejiang	3649	743	848	1591	11.9%	30.4%
	Fujian	2065	736	583	1319	15.9%	39.0%
	Shandong	4863	479	958	1437	5.6%	22.8%
	Guangdong	5647	1760	1758	3517	19.2%	38.4%
	Hainan	364	36	50	86	5.9%	19.1%
North-eastern China	Liaoning	1720	150	299	449	7.5%	20.7%
	Jilin	852	88	152	240	4.0%	22.0%
	Heilongjian	988	83	161	243	3.8%	19.8%
Central China	Shanxi	1205	97	147	244	2.8%	16.8%
	Anhui	2396	241	331	571	3.0%	19.3%
	Jiangxi	1456	285	280	565	6.4%	28.0%
	Henan	3732	355	394	750	2.7%	16.7%
	Hunan	2950	191	242	433	2.5%	12.8%
	Hubei	3000	278	445	723	5.6%	19.4%
Western China	Inner Mongolia	1263	93	133	227	1.4%	15.2%
	Guangxi	1390	188	202	389	6.3%	21.9%
	Chongqing	1466	241	299	540	9.3%	26.9%
	Sichuan	3275	196	320	516	5.1%	13.6%
	Guizhou	1104	109	147	256	2.0%	18.8%
	Yunnan	1659	90	99	189	3.5%	10.2%
	Shaanxi	1727	156	263	420	4.3%	19.6%
	Gansu	649	32	53	85	1.4%	11.6%
	Qinghai	229	8	9	17	1.6%	7.0%
	Ningxia	271	20	29	49	2.2%	15.3%
	Xinjiang	974	55	87	141	1.5%	12.7%

Contribution of MNEs to carbon emissions (1997 & 2002 & 2007 &2012 &2017) (Unit: million tons)

Regions	Provincial-level administrative entities	1997					
		Domestically-owned	HMT-owned	Foreign-owned	MNEs	Share of direct carbon emissions of MNEs in local carbon emissions	Contribution of MNEs to local carbon emissions
Eastern China	Beijing	36	7	12	19	10.7%	35.2%
	Tianjin	27	6	14	21	13.2%	43.5%
	Hebei	139	23	26	49	5.1%	26.1%
	Shanghai	52	16	30	46	9.5%	46.7%
	Jiangsu	95	28	54	82	19.5%	46.3%
	Zhejiang	81	14	16	30	9.0%	27.2%
	Fujian	19	14	9	22	21.2%	54.4%
	Shandong	144	18	32	50	9.2%	25.9%
	Guangdong	88	46	20	66	23.8%	42.8%
	Hainan	6	1	0	1	4.7%	15.0%
North-eastern China	Liaoning	127	21	43	64	7.9%	33.5%
	Jilin	76	6	12	18	2.7%	18.9%
	Heilongjiang	103	8	11	19	2.5%	15.7%
Central China	Shanxi	108	12	16	28	1.6%	20.7%
	Anhui	79	7	19	25	8.2%	24.2%
	Jiangxi	36	5	6	11	2.1%	23.2%
	Henan	110	13	13	26	4.8%	19.0%
	Hunan	71	7	10	17	4.9%	19.8%
	Hubei	89	21	13	34	10.7%	27.5%
Western China	Inner Mongolia	65	7	21	28	7.5%	30.4%
	Guangxi	41	4	4	8	3.2%	17.0%
	Chongqing	40	5	7	12	2.3%	22.8%
	Sichuan	91	5	11	16	1.7%	15.3%
	Guizhou	46	3	6	9	1.6%	16.5%
	Yunnan	41	3	6	9	3.5%	17.4%
	Shaanxi	50	5	6	11	0.8%	18.2%
	Gansu	37	3	4	7	0.5%	16.5%
	Qinghai	7	1	1	2	3.8%	23.2%
	Ningxia	11	1	4	5	1.1%	31.4%
	Xinjiang	47	3	4	7	1.2%	13.5%

Regions	Provincial-level administrative entities	2002					
		Domestically-owned	HMT-owned	Foreign-owned	MNEs	Share of direct carbon emissions of MNEs in local carbon emissions	Contribution of MNEs to local carbon emissions
Eastern China	Beijing	44	9	22	30	19.0%	40.9%
	Tianjin	45	7	20	27	8.7%	37.6%
	Hebei	210	25	49	74	7.4%	26.1%
	Shanghai	60	23	52	75	11.5%	55.4%
	Jiangsu	116	59	68	126	21.2%	52.2%
	Zhejiang	111	30	35	65	12.2%	36.9%
	Fujian	34	18	22	40	22.6%	54.2%
	Shandong	217	18	50	68	8.0%	23.8%
	Guangdong	127	61	49	110	15.6%	46.4%
	Hainan	8	1	4	5	15.2%	37.1%
North-eastern China	Liaoning	106	40	72	112	22.1%	51.4%
	Jilin	74	9	17	26	4.0%	26.1%
	Heilongjiang	94	9	21	30	2.8%	24.3%
Central China	Shanxi	164	14	44	58	12.5%	26.1%
	Anhui	112	17	24	41	7.9%	26.7%
	Jiangxi	52	5	7	11	4.6%	17.8%
	Henan	147	17	19	36	5.8%	19.6%
	Hunan	76	9	12	21	7.0%	21.3%
	Hubei	124	12	19	31	6.9%	20.2%
Western China	Inner Mongolia	104	12	20	32	3.4%	23.7%
	Guangxi	43	6	12	18	11.4%	29.9%
	Chongqing	53	9	11	19	7.7%	26.5%
	Sichuan	105	6	11	17	3.7%	13.6%
	Guizhou	64	5	8	14	2.4%	17.7%
	Yunnan	61	3	9	12	5.4%	16.6%
	Shaanxi	53	12	10	22	13.8%	29.4%
	Gansu	49	3	7	9	4.0%	16.2%
	Qinghai	12	1	1	2	2.1%	12.9%
	Ningxia	17	1	3	3	5.7%	17.1%
	Xinjiang	50	4	7	11	2.7%	17.6%
**Regions**	**Provincial-level administrative entities**	**2007**					
		**Domestically-owned**	**HMT-owned**	**Foreign-owned**	**MNEs**	**Share of direct carbon emissions of MNEs in local**	**Contribution of MNEs to local carbon**

						carbon emissions	emissions
Eastern China	Beijing	64	16	21	36	10.1%	36.0%
	Tianjin	74	10	26	36	7.1%	32.5%
	Hebei	343	76	124	199	8.8%	36.8%
	Shanghai	82	38	76	114	15.6%	58.2%
	Jiangsu	248	91	163	254	11.0%	50.6%
	Zhejiang	233	47	69	116	7.0%	33.2%
	Fujian	78	47	54	100	17.9%	56.2%
	Shandong	527	41	157	198	6.8%	27.3%
	Guangdong	235	111	91	202	11.6%	46.2%
	Hainan	19	2	3	5	1.1%	19.6%
North-eastern China	Liaoning	264	25	74	100	7.1%	27.4%
	Jilin	138	14	35	50	4.6%	26.4%
	Heilongjiang	151	13	34	46	3.5%	23.4%
Central China	Shanxi	269	20	52	72	5.7%	21.1%
	Anhui	163	27	45	72	5.1%	30.7%
	Jiangxi	104	14	16	30	3.2%	22.2%
	Henan	360	38	60	98	2.8%	21.4%
	Hunan	194	23	28	51	5.4%	20.8%
	Hubei	199	21	52	72	7.9%	26.6%
Western China	Inner Mongolia	265	35	57	92	4.1%	25.8%
	Guangxi	91	23	24	48	9.2%	34.3%
	Chongqing	82	10	16	26	9.4%	24.2%
	Sichuan	183	11	21	32	2.9%	14.8%
	Guizhou	136	18	24	42	2.8%	23.6%
	Yunnan	129	15	24	40	3.2%	23.5%
	Shaanxi	101	24	27	51	10.8%	33.4%
	Gansu	84	5	12	17	2.4%	16.7%
	Qinghai	24	1	1	3	1.2%	9.5%
	Ningxia	58	4	13	17	2.7%	22.6%
	Xinjiang	115	11	15	25	1.2%	18.0%
**Regions**	**Provincial-level administrative entities**	**2012**					
		**Domestically-owned**	**HMT-owned**	**Foreign-owned**	**MNEs**	**Share of direct carbon emissions of MNEs in local carbon emissions**	**Contribution of MNEs to local carbon emissions**
Eastern China	Beijing	63	12	14	25	8.6%	28.4%
	Tianjin	104	16	36	52	6.2%	33.4%
	Hebei	561	43	76	119	1.4%	17.5%

	Shanghai	90	37	67	104	16.9%	53.6%
	Jiangsu	362	93	168	260	7.7%	41.8%
	Zhejiang	241	59	63	122	5.7%	33.6%
	Fujian	135	56	43	99	11.4%	42.4%
	Shandong	665	53	140	193	3.1%	22.5%
	Guangdong	289	114	97	210	9.8%	42.1%
	Hainan	30	3	5	8	1.8%	21.2%
North-eastern China	Liaoning	380	37	70	108	3.2%	22.1%
	Jilin	200	13	19	32	2.6%	13.8%
	Heilongjiang	199	17	36	53	3.1%	21.0%
Central China	Shanxi	359	28	56	84	2.9%	19.0%
	Anhui	266	32	47	79	3.0%	22.8%
	Jiangxi	134	15	17	32	2.3%	19.3%
	Henan	400	40	59	99	2.3%	19.8%
	Hunan	243	18	24	42	1.8%	14.7%
	Hubei	316	18	41	59	4.6%	15.8%
Western China	Inner Mongolia	498	41	69	110	0.9%	18.1%
	Guangxi	167	18	20	38	3.3%	18.6%
	Chongqing	133	11	21	33	3.2%	19.7%
	Sichuan	274	15	26	41	2.9%	13.0%
	Guizhou	192	14	21	36	2.1%	15.7%
	Yunnan	166	15	26	42	3.8%	20.0%
	Shaanxi	203	15	30	45	2.3%	18.0%
	Gansu	121	8	16	24	2.2%	16.5%
	Qinghai	39	1	2	3	1.8%	8.1%
	Ningxia	107	10	16	25	0.7%	19.0%
	Xinjiang	210	21	27	48	0.2%	18.6%
**Regions**	**Provincial-level administrative entities**	**2017**					
		**Domestically-owned**	**HMT-owned**	**Foreign-owned**	**MNEs**	**Share of direct carbon emissions of MNEs in local carbon emissions**	**Contribution of MNEs to local carbon emissions**
Eastern China	Beijing	43	13	12	25	13.9%	36.9%
	Tianjin	89	14	29	43	3.8%	32.6%
	Hebei	500	67	119	186	7.4%	27.1%
	Shanghai	86	35	56	91	14.0%	51.4%
	Jiangsu	338	162	217	379	9.5%	52.8%
	Zhejiang	242	60	63	122	5.1%	33.6%
	Fujian	107	61	56	118	11.8%	52.4%
	Shandong	563	73	139	212	2.5%	27.4%

	Guangdong	300	111	90	201	10.9%	40.1%
	Hainan	31	3	6	10	7.0%	23.5%
North-eastern China	Liaoning	363	35	63	98	2.7%	21.3%
	Jilin	148	22	28	50	4.6%	25.1%
	Heilongjiang	193	22	44	66	4.9%	25.5%
Central China	Shanxi	375	40	55	96	1.4%	20.3%
	Anhui	256	47	52	100	4.6%	28.0%
	Jiangxi	138	35	43	78	3.8%	36.2%
	Henan	339	59	75	135	4.4%	28.4%
	Hunan	227	30	34	64	1.8%	22.0%
	Hubei	228	32	45	77	6.2%	25.3%
Western China	Inner Mongolia	492	54	81	135	1.4%	21.5%
	Guangxi	145	33	37	70	5.7%	32.4%
	Chongqing	106	19	23	42	3.5%	28.6%
	Sichuan	248	13	25	38	2.0%	13.2%
	Guizhou	183	25	31	56	1.5%	23.3%
	Yunnan	158	10	16	26	4.2%	14.3%
	Shaanxi	192	23	35	58	1.3%	23.1%
	Gansu	118	8	15	23	1.8%	16.3%
	Qinghai	43	3	4	7	1.9%	13.3%
	Ningxia	131	17	25	42	0.4%	24.3%
	Xinjiang	315	30	44	74	0.6%	19.0%

Supplementary Table 4 The four zones and 31 provincial-level administrative entities in China

Four zones in China	Provincial-level administrative entities
Eastern China	Beijing, Tianjin, Hebei, Shanghai, Jiangsu, Zhejiang, Fujian, Shandong, Guangdong, Hainan
Central China	Shanxi, Anhui, Jiangxi, Henan, Hubei, Hunan
Western China	Inner Mongolia, Guangxi, Chongqing, Sichuan, Guizhou, Yunnan, Shaanxi, Gansu, Qinghai, Ningxia, Xinjiang, Tibet
Northeastern China	Heilongjiang, Liaoning, Jilin

Note: China is officially divided into 34 province-level administrative divisions, namely 23 provinces, 5 autonomous regions, 4 municipalities directly under the central government, and 2 special administrative regions. Provinces: Anhui, Fujian, Gansu, Guangdong, Guizhou, Hainan, Hebei, Heilongjiang, Henan, Hubei, Hunan, Jiangsu, Jiangxi, Jilin, Liaoning, Qinghai, Shaanxi, Shandong, Shanxi, Sichuan, Yunnan, Zhejiang, and Taiwan; Autonomous Regions: Guangxi, Inner Mongolia, Ningxia, Xinjiang, Tibet; Municipalities: Beijing, Chongqing, Shanghai, Tianjin; Special Administrative Regions: Hong Kong, Macau.

Supplementary Table 5 Original and hypothetical C-Gini for China and four zones

**2017**	original	HMT	F	MNEs
China	0.320	0.327	0.333	0.343
Eastern China	0.268	0.276	0.282	0.286
North-eastern China	0.029	0.034	0.033	0.039
Central China	0.198	0.214	0.217	0.226
Western China	0.357	0.365	0.368	0.375
**2012**				
China	0.284	0.277	0.272	0.268
Eastern China	0.244	0.240	0.237	0.232
North-eastern China	0.037	0.038	0.043	0.046
Central China	0.159	0.154	0.151	0.146
Western China	0.288	0.280	0.278	0.272
**2007**				
China	0.253	0.243	0.235	0.231
Eastern China	0.228	0.228	0.212	0.211
North-eastern China	0.065	0.064	0.064	0.063
Central China	0.125	0.120	0.118	0.113
Western China	0.252	0.245	0.240	0.233
**2002**				
China	0.232	0.228	0.223	0.218
Eastern China	0.183	0.180	0.179	0.175
North-eastern China	0.042	0.048	0.052	0.058
Central China	0.226	0.219	0.217	0.211
Western China	0.198	0.190	0.192	0.184
**1997**				
China	0.221	0.212	0.209	0.203
Eastern China	0.171	0.173	0.176	0.160

North-eastern China	0.042	0.043	0.046	0.048
Central China	0.177	0.178	0.181	0.168
Western China	0.200	0.200	0.180	0.180

Note: ‘original’ presents the original C-Gini coefficients. ‘HMT’ and ‘F’ present the hypothetical C-Gini after extracting Hong Kong, Macao, Taiwan- (HMT-) owned enterprises and foreign-owned enterprises, respectively. ‘MNEs’ presents the hypothetical C-Gini after extracting both HMT-owned and foreign-owned enterprises.

Supplementary Table 6

Regression results of the effects of MNEs on emission coefficient

	(1)	(2)	(3)	(4)	(5.1)	(5.2)	(6)
	ec	*ec*	*ec*	*ec*	*MNE*	*ec*	*ec*
MNE	-0.012**	-0.017***	-0.017***	-0.017**		-0.023***	-0.017**
	(0.005)	(0.006)	(0.006)	(0.007)		(0.010)	(0.008)
sca		-0.004***	-0.004***	-0.004**	0.004***	-0.001***	0.001*
		(0.001)	(0.001)	(0.002)	(0.001)	(0.001)	(0.001)
str		0.006	0.006	0.006	-0.070*	-0.005*	-0.002
		(0.009)	(0.009)	(0.008)	(0.020)	(0.005)	(0.009)
tec		0.433***	0.433**	0.433**	0.720**	0.008**	0.107
		(0.163)	(0.197)	(0.139)	(0.266)	(0.085)	(0.103)
urb		-0.008*	-0.008*	-0.008*	0.124***	-0.009*	-0.008*
		(0.010)	(0.010)	(0.009)	(0.014)	(0.007)	(0.007)
reg		-0.003*	-0.003*	-0.003*	0.039***	0.003*	-0.002*
		(0.003)	(0.003)	(0.002)	(0.013)	(0.005)	(0.003)
constant	0.010***	0.029***	0.029***	0.029***	0.036***	0.015***	0.001
	(0.001)	(0.005)	(0.005)	(0.009)	(0.011)	(0.004)	(0.006)
FMA					-0.007***		
					(0.001)		
MNE (-5)					0.312***		
					(0.033)		
ec (-5)							0.481***
							(0.100)
Fixed Effect	Y	Y	Y	Y	Y	Y	Y
AR (1)							[0.004]
AR (2)							[0.105]
Overidentification test							[0.901]
Observations	3600	3023	3023	3023	2615	2615	2615
R^2	0.263	0.316	0.316	0.316	0.728	0.376	

Regression results of the effects of MNEs on emission coefficient gap

	(1)	(2)	(3)	(4)	(5.1)	(5.2)	(6)
	gap	*gap*	*gap*	*gap*	*MNE*	*gap*	*gap*
MNE	-2.409***	-1.818***	-1.818***	-1.818***		-7.893**	-7.280***
	(0.860)	(1.065)	(1.267)	(1.292)		(3.797)	(2.181)
sca		0.390***	0.390***	0.390***	0.004***	0.199**	0.139
		(0.092)	(0.102)	(0.155)	(0.001)	(0.108)	(0.129)
str		0.203	0.203	0.203	-0.021***	0.336*	4.305
		(1.520)	(1.661)	(1.266)	(0.020)	(2.172)	(3.032)
tec		-36.503**	-36.505**	-36.503**	0.720**	-45.905**	-44.156
		(20.406)	(18.708)	(18.007)	(0.266)	(24.292)	(35.622)
urb		0.096	0.096	0.096	0.124***	6.647**	3.263
		(1.222)	(0.996)	(1.053)	(0.014)	(2.870)	(2.531)
reg		-0.151*	-0.151*	-0.151*	0.039***	0.910*	-0.442
		(0.719)	(0.858)	(0.559)	(0.012)	(0.785)	(1.453)
constant	2.146***	0.5279**	0.5279*	0.5279*	0.036***	0.477**	-0.613
	(0.097)	(0.639)	(0.768)	(0.817)	(0.011)	(0.889)	(2.023)
FMA					-0.009***		
					(0.001)		
MNE (-5)					0.312***		
					(0.030)		
gap (-5)							0.390***
							(0.112)
Fixed Effect	Y	Y	Y	Y	Y	Y	Y
AR (1)							[0.000]
AR (2)							[0.108]
Overidentification test							[0.388]
Observations	3600	3023	3023	3023	2615	2615	2615
R^2	0.298	0.475	0.475	0.475	0.726	0.468	

Supplementary References

1 Chen, Q. *et al.* An interprovincial input–output database distinguishing firm ownership in China from 1997 to 2017. *Scientific Data* 10, 293 (2023).

2 Shan, Y. *et al.* China CO2 emission accounts 1997–2015. *Scientific data* 5, 1-14 (2018).

3 Shan, Y., Huang, Q., Guan, D. & Hubacek, K. China CO2 emission accounts 2016–2017. *Scientific data* 7, 54 (2020).

4 Zhang, Z. *et al.* Embodied carbon emissions in the supply chains of multinational enterprises. *Nature Climate Change* 10, 1096-1101 (2020).

5 Dietzenbacher, E., Pei, J. & Yang, C. Trade, production fragmentation, and China's carbon dioxide emissions. *Journal of Environmental Economics and Management* 64, 88-101 (2012).

6 Su, B. & Thomson, E. China's carbon emissions embodied in (normal and processing) exports and their driving forces, 2006–2012. *Energy Economics* 59, 414-422 (2016).

7 Cherniwchan, J., Copeland, B. R. & Taylor, M. S. Trade and the environment: New methods, measurements, and results. *Annual Review of Economics* 9, 59-85 (2017).

8 Lin, K., Tian, K., Gao, X., Zhao, Y. & Yang, C. Responses of China's cross-border investors to domestic environmental regulations. *Energy Economics* 131, 107371 (2024).

9 Koopman, R., Wang, Z. & Wei, S.-J. Tracing value-added and double counting in gross exports. *American economic review* 104, 459-494 (2014).

10 Los, B., Timmer, M. P. & De Vries, G. J. Tracing value-added and double counting in gross exports: Comment. *American Economic Review* 106, 1958-1966 (2016).
